# Frequency conversion in ultrastrong cavity QED

**DOI:** 10.1038/s41598-017-04225-3

**Published:** 2017-07-13

**Authors:** Anton Frisk Kockum, Vincenzo Macrì, Luigi Garziano, Salvatore Savasta, Franco Nori

**Affiliations:** 1 Center for Emergent Matter Science, Riken, Saitama 351-0198 Japan; 2 Dipartimento di Scienze Matematiche e Informatiche, Scienze Fisiche e Scienze della Terra, Università di Messina, I-98166 Messina, Italy; 3 School of Physics and Astronomy, University of Southampton, Southampton, SO17 1BJ United Kingdom; 4 Physics Department, The University of Michigan, Ann Arbor, Michigan 48109-1040 USA

## Abstract

We propose a new method for frequency conversion of photons which is both versatile and deterministic. We show that a system with two resonators ultrastrongly coupled to a single qubit can be used to realise both single- and multiphoton frequency-conversion processes. The conversion can be exquisitely controlled by tuning the qubit frequency to bring the desired frequency-conversion transitions on or off resonance. Considering recent experimental advances in ultrastrong coupling for circuit QED and other systems, we believe that our scheme can be implemented using available technology.

## Introduction

Frequency conversion in quantum systems^1, 2^, is important for many quantum technologies. The optimal working points of devices for transmission, detection, storage, and processing of quantum states are spread across a wide spectrum of frequencies^3, 4^. Interfacing the best of these devices is necessary to create quantum networks^5^ and other powerful combinations of quantum hardware. Examples of frequency-conversion setups developed for such purposes include upconversion for photon detection^6^ and storage^7^, since both these things are easier to achieve at a higher frequency than what is optimal for telecommunications. Downconversion in this frequency range has also been demonstrated^8–10^, and recently even strong coupling between a telecom and a visible optical mode^11^. Additionally, advances in quantum information processing with superconducting circuits at microwave frequencies^12, 13^, is driving progress on frequency conversion between optical and microwave frequencies^14–17^. We note that several types of quantum systems, suited for different tasks in quantum information processing, can operate at microwave frequencies^4^. To connect these systems, frequency conversion within this frequency range is important. Furthermore, frequency conversion can be used to create entangled states, which have applications in virtually all areas of quantum information processing, including quantum computing, quantum key distribution, and quantum teleportation^18^.

Circuit quantum electrodynamics (QED)^12, 19–22^, offers a wealth of possibilities for frequency conversion at microwave frequencies; some of these schemes can also be generalised to optical frequencies. By modulating the magnetic flux through a superconducting quantum interference device (SQUID) in a transmission-line resonator, the frequency of the photons in the resonator can be changed rapidly^23–25^, or two modes of the resonator can be coupled^26, 27^. Other driven Josephson-junction-based devices can also be used for microwave frequency conversion^28, 29^. Downconversion has been proposed for setups with Δ-type three-level atoms^30–32^, and demonstrated with an effective three-level Λ system^33^. Upconversion of a two-photon drive has been shown for a flux qubit coupled to a resonator in a way that breaks parity symmetry^34^. Indeed, the Δ-type level structure in a flux qutrit^35^ even makes possible general three-wave mixing^36^. Recently, frequency conversion was also demonstrated for two sideband-driven microwave *LC*-resonators coupled through a mechanical resonator^37^.

The approach to frequency conversion that we propose in this article is based on two cavities or resonator modes coupled ultrastrongly to a two-level atom (qubit). The regime of ultrastrong coupling (USC), where the coupling strength starts to become comparable to the bare transition frequencies in the system, has only recently been reached in a number of solid-state systems^38–56^. Among these, a few circuit-QED experiments provide some of the clearest examples^39, 40, 49–52, 54–56^, including the largest coupling strength reported^51^. While the USC regime displays many striking physical phenomena^57–66^, we are here only concerned with the fact that it enables higher-order processes that do not conserve the number of excitations in the system, an effect which has also been noted for a multilevel atom coupled to a resonator^67^. Examples of such processes include multiphoton Rabi oscillations^68, 69^, and a single photon exciting multiple atoms^70^. Indeed, almost any analogue of processes from nonlinear optics is feasible^71^; this can be regarded as an example of quantum simulation^72, 73^. Just like the analytical solution for the quantum Rabi model^74^ is now being extended to multiple qubits^75, 76^, and multiple resonators^77–79^, we here extend the exploration of non-excitation-conserving processes to multiple resonators.

In our proposal, the qubit frequency is tuned to make various frequency-converting transitions resonant. For example, making the energy of a single photon in the first resonator equal to the sum of the qubit energy and the energy of a photon in the second resonator enables the conversion of the former (a high-energy photon) into the latter (a low-energy photon plus a qubit excitation) and vice versa. In the same way, a single photon in the first resonator can be converted into multiple photons in the second resonator (and vice versa) if the qubit energy is tuned to make such a transition resonant. The proposed frequency-conversion scheme is deterministic and allows for a variety of different frequency-conversion processes in the same setup. The setup should be possible to implement in state-of-the-art circuit QED, but the idea also applies to other cavity-QED systems.

We note that the process of parametric down-conversion in this type of circuit-QED setup has been considered previously^80^, but in a regime of weaker coupling and without using the qubit to control the process. Also, it has been shown that a beamsplitter-type coupling between two resonators can be controlled by changing the qubit state^81^ or induced for weaker qubit-resonator coupling by driving the qubit^82^, but the proposal presented here offers greater versatility and simplicity for frequency conversion.

## Model

**Figure 1:**
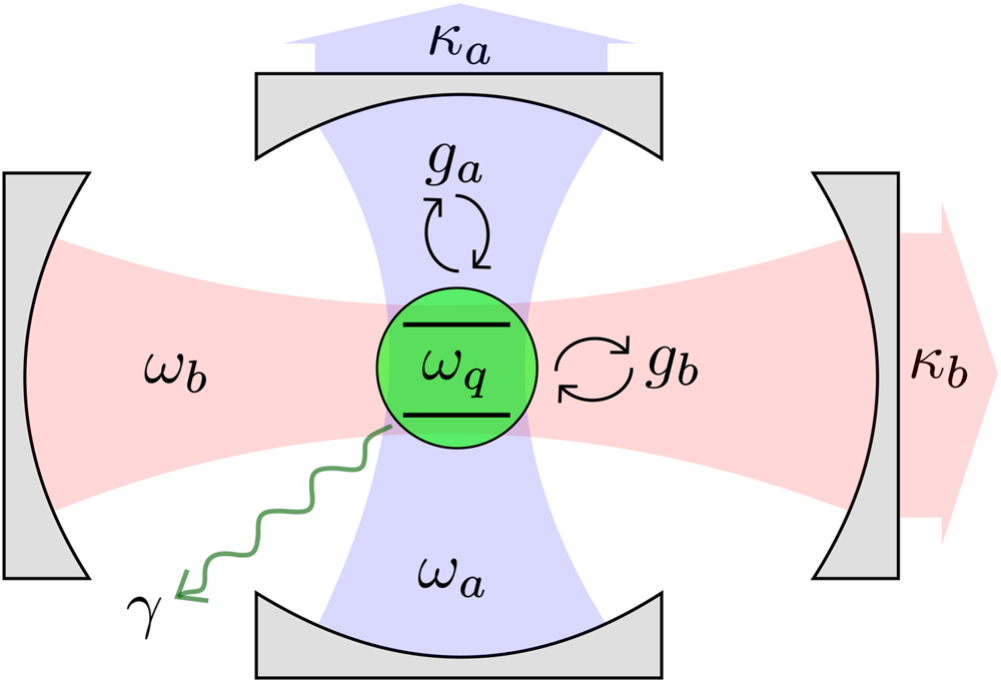
A sketch of the system. A qubit (green) is coupled to two resonator modes (blue, *a*, and red, *b*). Decoherence channels for the qubit (relaxation rate *γ*) and the resonators (relaxation rates *κ*_*a*_, *κ*_*b*_) are included.

We consider a setup where a qubit with transition frequency *ω*_*q*_ is coupled to two resonators with resonance frequencies *ω*_*a*_ and *ω*_*b*_, respectively, as sketched in Fig. 1. The Hamiltonian is (*ħ* = 1)

1 $$\hat{H}={\omega }_{a}{\hat{a}}^{\dagger }\hat{a}+{\omega }_{b}{\hat{b}}^{\dagger }\hat{b}+\frac{{\omega }_{q}}{2}{\hat{\sigma }}_{z}+[{g}_{a}(\hat{a}+{\hat{a}}^{\dagger })+{g}_{b}(\hat{b}+{\hat{b}}^{\dagger })]\,({\hat{\sigma }}_{x}\,\cos \,\theta +{\hat{\sigma }}_{z}\,\sin \,\theta ),$$

where *g*_*a*_ (*g*_*b*_) denotes the strength of the coupling between the qubit and the first (second) resonator. The creation and annihilation operators for photons in the first (second) resonator are $\hat{a}$ and ${\hat{a}}^{\dagger }$ ($\hat{b}$ and ${\hat{b}}^{\dagger }$), respectively. The angle *θ* parameterises the amount of longitudinal and transverse coupling as, for example, in experiments with flux qubits^34, 39, 40, 49, 56, 83^; ${\hat{\sigma }}_{x}$ and ${\hat{\sigma }}_{z}$ are Pauli matrices for the qubit.

Note that we do not include a direct coupling between the two resonators. Such a coupling is seen in experiments^49^, but here we will only be concerned with situations where the resonators are far detuned from each other, meaning that this coupling term can safely be neglected. Likewise, we do not include higher modes of the resonators. While they may contribute in experiments with cavities and transmission-line resonators, they can be avoided by using lumped-element resonators^56, 84^.

The crucial feature of Eq. (1) for our frequency-conversion scheme is that some of the coupling terms do not conserve the number of excitations in the system. The ${\hat{\sigma }}_{z}$ coupling terms act to change the photon number in one of the resonators by one, while keeping the number of qubit excitations unchanged. Likewise, the ${\hat{\sigma }}_{x}$ coupling contains terms like $\hat{a}{\hat{\sigma }}_{-}$ and ${\hat{b}}^{\dagger }{\hat{\sigma }}_{+}$ that change the number of excitations in the system by two. For weak coupling strengths, all such terms can be neglected using the rotating-wave approximation (RWA), but in the USC regime the higher-order processes that these terms enable can become important and function as second- or third-order nonlinearities in nonlinear optics^71^.

To include the effect of decoherence in our system, we use a master equation on the Lindblad form in our numerical simulations. The master equation reads

2 $$\dot{\hat{\rho }}=-i\,[\hat{H},\hat{\rho }]+\sum _{j,k > j}({{\rm{\Gamma }}}_{a}^{jk}+{{\rm{\Gamma }}}_{b}^{jk}+{{\rm{\Gamma }}}_{q}^{jk}){\mathscr{D}}[|j\rangle \langle k|]\hat{\rho },$$

where $\hat{\rho }$ is the density matrix of the system, ${\mathscr{D}}[\hat{c}]\rho =\hat{c}\hat{\rho }{\hat{c}}^{\dagger }-\frac{1}{2}\hat{\rho }{\hat{c}}^{\dagger }\hat{c}-\frac{1}{2}{\hat{c}}^{\dagger }\hat{c}\hat{\rho }$, and the states in the sum are eigenstates of the USC system. The relaxation rates are given by ${{\rm{\Gamma }}}_{a}^{jk}={\kappa }_{a}{|{X}_{a}^{jk}|}^{2}$, ${{\rm{\Gamma }}}_{b}^{jk}={\kappa }_{b}{|{X}_{b}^{jk}|}^{2}$, and ${{\rm{\Gamma }}}_{q}^{jk}=\gamma {|{C}^{jk}|}^{2}$, where *κ*_*a*_, *κ*_*b*_, and *γ* are the relaxation rates for the bare states of the resonators and the qubit, respectively, and ${c}^{jk}=\langle \,j|\hat{c}|k\rangle$ with ${\hat{X}}_{a}=\hat{a}+{\hat{a}}^{\dagger }$, ${\hat{X}}_{b}=\hat{b}+{\hat{b}}^{\dagger }$, and $\hat{C}={\hat{\sigma }}_{x}$ ^85, 86^, Writing the master equation in the eigenbasis of the full system avoids unphysical effects, such as emission of photons from the ground state. Similarly, to correctly count the number of photonic and qubit excitations we use $\langle {\hat{X}}_{a}^{-}{\hat{X}}_{a}^{+}\rangle$, $\langle {\hat{X}}_{b}^{-}{\hat{X}}_{b}^{+}\rangle$, and $\langle {\hat{C}}^{-}{\hat{C}}^{+}\rangle$, where the plus and minus signs denote the positive and negative frequency parts, respectively, of the operators in the system eigenbasis, instead of $\langle {\hat{a}}^{\dagger }\hat{a}\rangle$, $\langle {\hat{b}}^{\dagger }\hat{b}\rangle$, and $\langle {\hat{\sigma }}_{+}{\hat{\sigma }}_{-}\rangle$ ^86^.

In the simulations presented in the next section, we use parameters that can be reached in circuit-QED experiments. In such experiments, the bare transition frequencies are usually in the range *ω*_*a*/*b*/*q*_ ~ 2*π* × 1 − 10 GHz. When it comes to coupling strengths, several experiments have demonstrated *g*_*a*/*b*_ ≳ 0.1*ω*_*a*/*b*_^39, 40, 49, 52^, and recently even *g*_*a*/*b*_ ~ *ω*_*a*/*b*_ was reached^51, 56^. In all these experiments, superconducting flux qubits are coupled either to lumped-element *LC* oscillators^39, 51, 56^, or transmission-line resonators^40, 49, 52^. For transmission-line resonators, quality factors *Q* = *ω*_*a*/*b*_/*κ*_*a*/*b*_ exceeding 10^6^ have been demonstrated^87^, and flux qubit relaxation rates *γ* can now be as small as ~2*π* × 10 kHz^88–90^. This brief survey of parameters indicates that *γ*,*κ*_*a*/*b*_ ~ 10^−6^ *ω*_*a*/*b*/*q*_ is possible and that *g*_*a*/*b*_ can be a large fraction of *ω*_*a*/*b*/*q*_ if needed. In the numerical simulations for different frequency-conversion processes, we choose more conservative values for the decoherence rates (more than an order of magnitude larger than the best values discussed here), at the same time restricting the coupling strengths *g*_*a*/*b*_ to as small values as possible (10–20% of the qubit frequency, depending on the setup) while still achieving high conversion efficiency. We note that the *g*_*a*/*b*_ values we chose make the coupling ultrastrong with respect to *ω*_*q*_, but not ultrastrong with respect to *ω*_*a*/*b*_.

## Results

### Single-photon frequency conversion

We first consider single-photon frequency conversion, where one photon in the first resonator is converted into one photon of a different frequency in the second resonator, or vice versa. The conversion is aided by the qubit. Without loss of generality, we take *ω*_*a*_ > *ω*_*b*_. For the conversion to work, we then need *ω*_*a*_ ≈ *ω*_*b*_ + *ω*_*q*_, such that the states |1, 0, *g*〉 and |0, 1, *e*〉 are close to resonant. Due to the presence of longitudinal coupling in the Hamiltonian in Eq. (1), transitions between these two states are possible even though their excitation numbers and parity differ.

**Figure 2:**
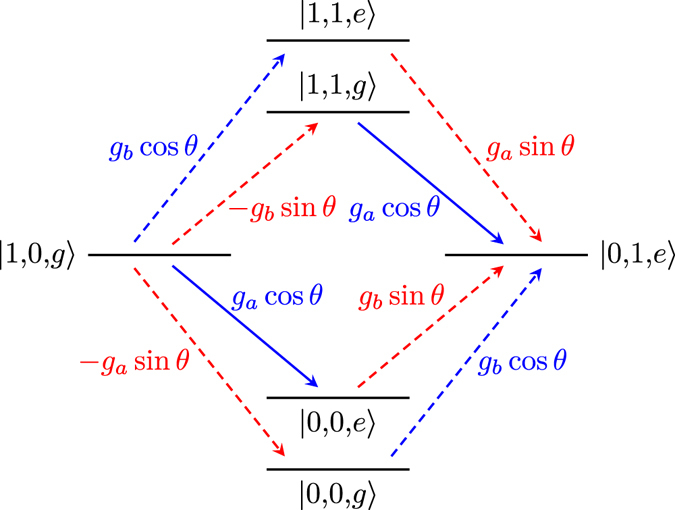
The four lowest-order processes contributing to a transition between |1, 0, *g*〉 and |0, 1, *e*〉. For this illustration, the parameter values *ω*_*a*_ = 3*ω*_*q*_ and *ω*_*b*_ = 2*ω*_*q*_ were used to set the positions of the energy levels. The transitions that do not conserve excitation number are shown as dashed lines, and the excitation-number-conserving transitions are shown as solid lines. Red lines correspond to ${\hat{\sigma }}_{z}$ (longitudinal) coupling and blue lines to ${\hat{\sigma }}_{x}$ (transverse) coupling in the Hamiltonian given in Eq. (1). Each transition is labelled by its matrix element.

The intermediate states and transitions contributing (in lowest order) to the |1, 0, *g*〉 ↔ |0, 1, *e*〉 transition are shown in Fig. 2. Virtual transitions to and from one of the four intermediate states |0, 0, *g*〉, |0, 0, *e*〉, |1, 1, *g*〉, and |1, 1, *e*〉 connect |1, 0, *g*〉 and |0, 1, *e*〉 in two steps. This is the minimum number of steps possible, since the terms in the Hamiltonian in Eq. (1) can only create or annihilate a single photon at a time. From the figure, it is also clear that no path exists between |1, 0, *g*〉 and |0, 1, *e*〉 that does not involve longitudinal coupling (dashed red arrows in the figure).

To calculate the effective coupling between the states |1, 0, *g*〉 and |0, 1, *e*〉, we truncate the Hamiltonian from Eq. (1) to the six states shown in Fig. 2. Written on matrix form, this truncated Hamiltonian becomes

3 $$\hat{H}=(\begin{array}{cccccc}-\frac{{\omega }_{q}}{2} & 0 & -{g}_{a}\,\sin \,\theta  & {g}_{b}\,\cos \,\theta  & 0 & 0\\ 0 & \frac{{\omega }_{q}}{2} & {g}_{a}\,\cos \,\theta  & {g}_{b}\,\sin \,\theta  & 0 & 0\\ -{g}_{a}\,\sin \,\theta  & {g}_{a}\,\cos \,\theta  & {\omega }_{a}-\frac{{\omega }_{q}}{2} & 0 & -{g}_{b}\,\sin \,\theta  & {g}_{b}\,\cos \,\theta \\ {g}_{b}\,\cos \,\theta  & {g}_{b}\,\sin \,\theta  & 0 & {\omega }_{b}+\frac{{\omega }_{q}}{2} & {g}_{a}\,\cos \,\theta  & {g}_{a}\,\sin \,\theta \\ 0 & 0 & -{g}_{b}\,\sin \,\theta  & {g}_{a}\,\cos \,\theta  & {\omega }_{a}+{\omega }_{b}-\frac{{\omega }_{q}}{2} & 0\\ 0 & 0 & {g}_{b}\,\cos \,\theta  & {g}_{a}\,\sin \,\theta  & 0 & {\omega }_{a}+{\omega }_{b}+\frac{{\omega }_{q}}{2}\end{array}),$$

where the states are ordered from left to right as |0, 0, *g*〉, |0, 0, *e*〉, |1, 0, *g*〉, |0, 1, *e*〉, |1, 1, *g*〉, and |1, 1, *e*〉. When the condition *ω*_*a*_ ≈ *ω*_*b*_ + *ω*_*q*_ is satisfied, the four intermediate states |0, 0, *g*〉, |0, 0, *e*〉, |1, 1, *g*〉, and |1, 1, *e*〉 can be adiabatically eliminated, i.e., provided that the bare coupling strengths are sufficiently small compared to the energy difference between the intermediate states and the initial and final states, we can assume that the population of the intermediate states will not change significantly, such that the effective dynamics will only involve the initial and final states. This calculation, shown in the Supplementary Information, gives an effective Hamiltonian with a coupling term

4 $${\hat{H}}_{{\rm{c}},{\rm{eff}}}={g}_{{\rm{eff}}}(|\mathrm{1,}\,\mathrm{0,}\,g\rangle \langle \mathrm{0,}\,\mathrm{1,}\,e|+|\mathrm{0,}\,\mathrm{1,}\,e\rangle \langle \mathrm{1,}\,\mathrm{0,}\,g|),$$

where the effective coupling between the states |1, 0, *g*〉 and |0, 1, *e*〉 has the magnitude

5 $${g}_{{\rm{eff}}}={g}_{a}{g}_{b}\,\sin \,2\theta \,(\frac{1}{{\omega }_{b}}-\frac{1}{{\omega }_{a}})$$

on resonance. Compared to the direct resonator-qubit coupling in Eq. (1), *g*_eff_ is weaker by a factor of order *g*/*ω*, which is why we need to at least approach the USC regime to observe the single-photon frequency conversion. We note that the effective coupling is maximised when the longitudinal and transverse coupling terms in Eq. (1) have equal magnitude. Interestingly, Eq. (5) suggests that frequency conversion can be more efficient if *ω*_*b*_ ≪ *ω*_*a*_. However, going too far in this direction violates the assumptions behind the adiabatic approximation, which relies on *g*_*a*_,*g*_*b*_ ≪ *ω*_*a*_,*ω*_*b*_.

**Figure 3:**
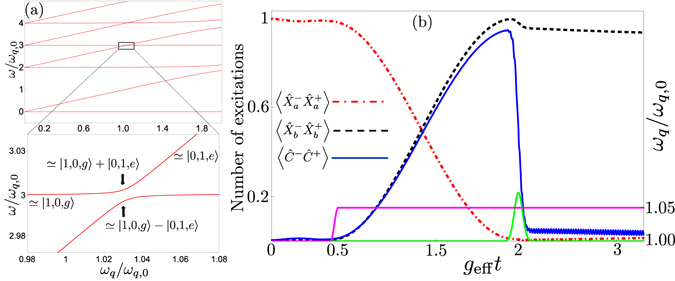
Two frequency conversion methods. (a) The figure shows the energy levels of our system plotted as a function of the qubit frequency *ω*_*q*_, using the parameters *g*_*a*_ = *g*_*b*_ = 0.15*ω*_*q*,0_, *θ* = *π*/6, *ω*_*a*_ = 3*ω*_*q*,0_, and *ω*_*b*_ = 2*ω*_*q*,0_, where *ω*_*q*,0_ is a reference point for the qubit frequency, set such that *ω*_*a*_ = *ω*_*b*_ + *ω*_*q*,0_. In the zoom-in, close to the resonance *ω*_*a*_ = *ω*_*b*_ + *ω*_*q*_, we see the anticrossing between |1, 0, *g*〉 and |0, 1, *e*〉 with splitting 2*g*_eff_. Up- or down-conversion of single photons can be achieved by adiabatically tuning *ω*_*q*_ to follow one of the energy levels in the figure from |1, 0, *g*〉 to |0, 1, *e*〉, or vice versa. (b) A rapid frequency conversion can be achieved by starting in |1, 0, *g*〉, far from the resonance *ω*_*a*_ = *ω*_*b*_ + *ω*_*q*_, tuning the qubit frequency (pink solid curve) into resonance for half a Rabi period (*π*/2*g*_eff_) and then sending a pulse (green solid curve) to deexcite the qubit. The pink solid curve is given by ${\omega }_{q}(t)={\omega }_{q,i}+\delta {\omega }_{q}\{{\sin }^{2}[A(t-{t}_{i})]{\rm{\Theta }}(t-{t}_{i})+{\sin }^{2}[A(t-{t}_{f})]{\rm{\Theta }}(t-{t}_{f})\}$, a smoothed step function, where *ω*_*q*,*i*_ is the initial qubit frequency, *δω*_*q*_ is the change of the qubit frequency, Θ is the Heaviside step function, *t*_*i*_ is the time when the qubit frequency starts to change, *t*_*f*_ = *t*_*i*_ + *π*/(2*A*), and *A* is a frequency setting the smoothness. The figure shows the number of excitations in the two resonators (red dashed-dotted curve for *a*, black dashed curve for *b*) and the qubit (blue solid curve) during the process, including decoherence in the form of relaxation from the resonators and the qubit. The parameters used for the decoherence are *κ*_*a*_ = *κ*_*b*_ = *γ* = 4 × 10^−5^ *ω*_*q*,0_.

The existence of this effective coupling suggests at least two ways to perform single-photon frequency conversion. The first is to use adiabatic transfer, starting in |1, 0, *g*〉 (|0, 1, *e*〉) with the qubit frequency sufficiently far detuned from the resonance *ω*_*a*_ = *ω*_*b*_ + *ω*_*q*_ and then slowly [adiabatically, i.e., slow enough that the probability of a Landau-Zener transition back to the initial state is small; note that this is a different notion of adiabaticity than that used in the adiabatic elimination used to derive Eq. (4)] changing the qubit frequency until the system ends up in the state |0, 1, *e*〉 (|1, 0, *g*〉), following one of the energy levels shown in Fig. 3(a). In this way, a single photon in the first (second) resonator is deterministically down-converted (up-converted) to a single photon of lower (higher) frequency in the second (first) resonator. We note that such adiabatic transfer has been used for robust single-photon generation in circuit QED, tuning the frequency of a transmon qubit to achieve the transition |0, *e*〉 → |1, *g*〉^91^. It has also been suggested as a method to generate multiple photons from a single qubit excitation in the USC regime of the standard quantum Rabi model^68^.

The second approach, exemplified by a simulation including decoherence in Fig. 3(b), is to initialise the system in one of the states |1, 0, *g*〉 or |0, 1, *e*〉, far from the frequency-conversion resonance such that the effective coupling is negligible, quickly tune the qubit into resonance for the duration of half a Rabi oscillation period (set by the effective coupling to be *π*/2*g*_eff_), and then detune the qubit again (or send a pulse to deexcite it) to turn off the effective interaction. This type of scheme is, for example, commonly used for state transfer between resonators and/or qubits in circuit QED^92–96^. Letting the resonance last shorter or longer times, any superposition of |1, 0, *g*〉 or |0, 1, *e*〉 can be created. The potential for creating superpositions of photons of different frequencies (similar to ref. 27) with such a method will be explored in future work.

**Figure 4:**
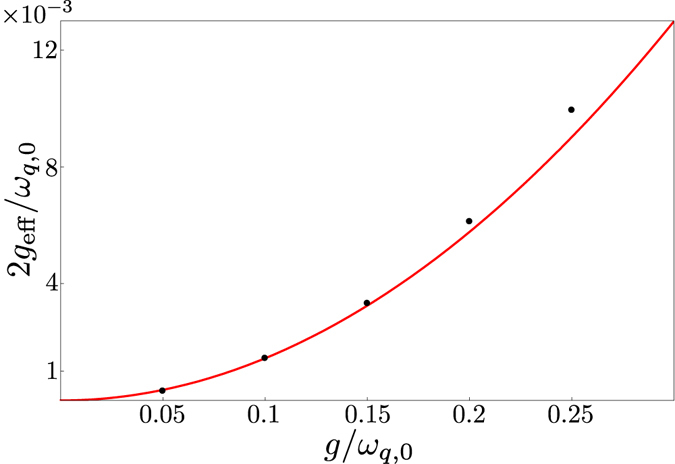
Comparison of analytical (red curve) and numerical (black dots) results for the effective coupling between the states |1, 0, *g*〉 and |0, 1, *e*〉. The graph shows the minimum energy splitting 2*g*_eff_/*ω*_*q*,0_ as a function of *g*/*ω*_*q*,0_, where *g* = *g*_*a*_ = *g*_*b*_, using the same parameters as in Fig. 3.

Since the relevant timescales for both these approaches are determined by *g*_eff_, it is important to know in which parameter range the expression for *g*_eff_ given in Eq. (5) remains a good approximation. In Fig. 4, we show that the expression is valid up to at least *g*_*a*_ = *g*_*b*_ = 0.2*ω*_*q*,0_ for the parameters used in Fig. 3.

We note that both protocols for frequency-conversion given here can be used to transfer superposition states. For example, starting in the superposition state *a*|0, 0, *g*〉 + *b*|1, 0, *g*〉 = (*a*|0〉 + *b*|1〉)|0, *g*〉, where *a* and *b* are complex numbers satisfying |*a*|^2^ + |*b*|^2^ = 1, both protocols will convert this state into *a*|0, 0, *g*〉 + *b*|0, 1, *e*〉 = |0〉(*a*|0, *g*〉 + *b*|1, *e*〉). If one wishes to disentangle the qubit from the second resonator mode after the transfer of the superposition, a photon-number-dependent qubit rotation, which can be implemented in the strong-dispersive regime of circuit QED^97^, is one option. The remarks given here also apply to the multi-photon frequency-conversion processes studied in the next section.

### Multi-photon frequency conversion

We now turn to multi-photon frequency conversion, where, aided by the qubit, one photon in the first resonator is converted into two photons in the second resonator, or vice versa. We continue to adopt the convention that *ω*_*a*_ > *ω*_*b*_. In contrast to the single-photon frequency conversion case above, there are now two possibilities for how the qubit state can change during the conversion process. Below, we will study both |1, 0, *g*〉 ↔ |0, 2, *e*〉 and |1, 0, *e*〉 ↔ |0, 2, *g*〉. Since we wish to use the qubit to control the process, we do not consider the process |1, 0, *g*〉 ↔ |0, 2, *g*〉, which to some extent was already included in ref. 80, although that work considered a setup with *ω*_*q*_ ≈ *ω*_*b*_ and mainly studied the squeezing produced by a strong external drive.

### The |1, 0, *g*〉 ↔ |0,2,*e*〉 process

For the process |1, 0, *g*〉 ↔ |0, 2, *e*〉, we first of all note one more difference compared to the single-photon frequency conversion case: it changes the number of excitations from 1 to 3, which means that excitation-number parity is conserved. This makes the longitudinal coupling of Eq. (1) redundant for achieving the conversion, and to simplify our calculations we therefore hereafter work with the standard quantum Rabi Hamiltonian^98^ extended to two resonators,

6 $${\hat{H}}_{{\rm{R}}}={\omega }_{a}{\hat{a}}^{\dagger }\hat{a}+{\omega }_{b}{\hat{b}}^{\dagger }\hat{b}+\frac{{\omega }_{q}}{2}{\hat{\sigma }}_{z}+[{g}_{a}(\hat{a}+{\hat{a}}^{\dagger })+{g}_{b}(\hat{b}+{\hat{b}}^{\dagger })]{\hat{\sigma }}_{x}.$$

**Figure 5:**
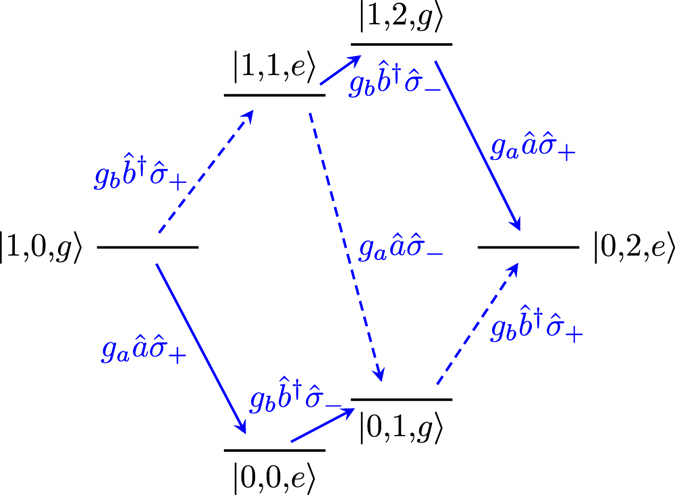
The lowest-order processes contributing to a transition between |1, 0, *g*〉 and |0, 2, *e*〉 in the quantum Rabi model. The transitions that do not conserve excitation number are shown as dashed blue lines and the excitation-number-conserving transitions are shown as solid blue lines. The label of each line is the term in Eq. (6) that gives rise to that transition. The parameters *ω*_*a*_ = 5*ω*_*q*_ and *ω*_*b*_ = 2*ω*_*q*_ were used to set the positions of the energy levels.

Placing the system close to the resonance *ω*_*a*_ = 2*ω*_*b*_ + *ω*_*q*_, virtual transitions involving the intermediate states |0, 0, *e*〉, |0, 1, *g*〉, |1, 1, *e*〉, and |1, 2, *g*〉 (to lowest order), contribute to the process |1, 0, *g*〉 ↔ |0, 2, *e*〉, as shown in Fig. 5. The most direct path between |1, 0, *g*〉 and |0, 2, *e*〉 involves three steps, since only one photon can be created or annihilated in each step. We note that all the paths include at least one transition that is due to terms in the Hamiltonian that do not conserve excitation number (dashed arrows in the figure).

Retaining only the states shown in Fig. 5, we can write the quantum Rabi Hamiltonian from Eq. (6) on matrix form as

7 $${\hat{H}}_{{\rm{R}}}=(\begin{array}{cccccc}\frac{{\omega }_{q}}{2} & {g}_{b} & {g}_{a} & 0 & 0 & 0\\ {g}_{b} & {\omega }_{b}-\frac{{\omega }_{q}}{2} & 0 & \sqrt{2}{g}_{b} & {g}_{a} & 0\\ {g}_{a} & 0 & {\omega }_{a}-\frac{{\omega }_{q}}{2} & 0 & {g}_{b} & 0\\ 0 & \sqrt{2}{g}_{b} & 0 & 2{\omega }_{b}+\frac{{\omega }_{q}}{2} & 0 & {g}_{a}\\ 0 & {g}_{a} & {g}_{b} & 0 & {\omega }_{a}+{\omega }_{b}+\frac{{\omega }_{q}}{2} & \sqrt{2}{g}_{b}\\ 0 & 0 & 0 & {g}_{a} & \sqrt{2}{g}_{b} & {\omega }_{a}+2{\omega }_{b}-\frac{{\omega }_{q}}{2}\end{array}),$$

where the states are ordered as |0, 0, *e*〉, |0, 1, *g*〉, |1, 0, *g*〉, |0, 2, *e*〉, |1, 1, *e*〉, and |1, 2, *g*〉. Just like before, we can adiabatically eliminate the intermediate states when the condition *ω*_*a*_ ≈ 2*ω*_*b*_ + *ω*_*q*_ is satisfied. The result of this calculation, the details of which are given in the Supplementary Information, is an effective coupling between the states |1, 0, *g*〉 and |0, 2, *e*〉 with magnitude

8 $${g}_{{\rm{eff}}}=\frac{\sqrt{2}{g}^{3}[2{\omega }_{b}({\omega }_{a}-2{\omega }_{b})-{g}^{2}]}{2{\omega }_{b}^{2}{({\omega }_{a}-{\omega }_{b})}^{2}+{g}^{2}{\omega }_{b}\mathrm{(5}{\omega }_{b}-3{\omega }_{a})+{g}^{4}}$$

on resonance. Here, we have set *g*_*a*_ = *g*_*b*_ ≡ *g* to simplify the expression slightly. We note that, to leading order, the coupling scales like *g* ^3^/*ω* ^2^; indeed, the leading-order term is

9 $${g}_{{\rm{eff}}}=\frac{\sqrt{2}{g}^{3}({\omega }_{a}-2{\omega }_{b})}{{\omega }_{b}{({\omega }_{a}-{\omega }_{b})}^{2}}\mathrm{.}$$

This is a factor *g*/*ω* weaker than for the single-photon frequency conversion, and reflects the fact that an additional intermediate transition is required for the two-photon conversion. We also note that the coupling becomes small in the limit of small *ω*_*q*_, i.e., when 2*ω*_*b*_ → *ω*_*a*_. The coupling would become large if *ω*_*a*_ → *ω*_*b*_, but this is impossible since *ω*_*a*_ = 2*ω*_*b*_ + *ω*_*q*_ in this scheme.

**Figure 6:**
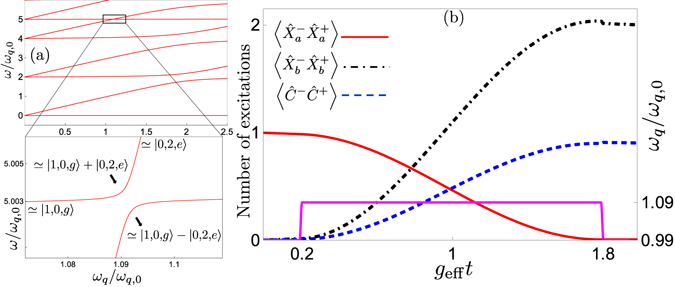
Two-photon frequency conversion via transitions between |1, 0, *g*〉 and |0, 2, *e*〉. (a) The energy levels of our system, given in Eq. (6), plotted as a function of the qubit frequency *ω*_*q*_, using the parameters *g*_*a*_ = *g*_*b*_ = 0.2*ω*_*q*,0_, *ω*_*a*_ = 5*ω*_*q*,0_, and *ω*_*b*_ = 2*ω*_*q*,0_, where the reference point *ω*_*q*,0_ is set such that *ω*_*a*_ = 2*ω*_*b*_ + *ω*_*q*,0_. In the zoom-in, close to the resonance *ω*_*a*_ = 2*ω*_*b*_ + *ω*_*q*_, we see the anticrossing between |1, 0, *g*〉 and |0, 2, *e*〉 with the splitting 2*g*_eff_ given by Eq. (8). Up-conversion of a photon pair into a single photon, or down-conversion of a single photon into a photon pair, can be achieved by adiabatically tuning *ω*_*q*_ to follow one of the energy levels in the figure from |0, 2, *e*〉 to |1, 0, *g*〉, or vice versa. (b) A rapid frequency conversion can be achieved by starting in |1, 0, *g*〉 or |0, 2, *e*〉, far from the resonance *ω*_*a*_ = 2*ω*_*b*_ + *ω*_*q*_, tuning the qubit frequency (pink solid curve, a smoothed step function as explained in Fig. 3) into resonance for half a Rabi period (*π*/2*g*_eff_) and then tuning it out of resonance again. The figure shows the number of excitations in the two resonators (red solid curve for *a*, black dashed-dotted curve for *b*) and the qubit (blue dashed curve) during such a process, including decoherence in the form of relaxation from the resonators and the qubit. The parameters used for the decoherence are *κ*_*a*_ = *κ*_*b*_ = *γ* = 2 × 10^−5^ *ω*_*q*,0_.

**Figure 7:**
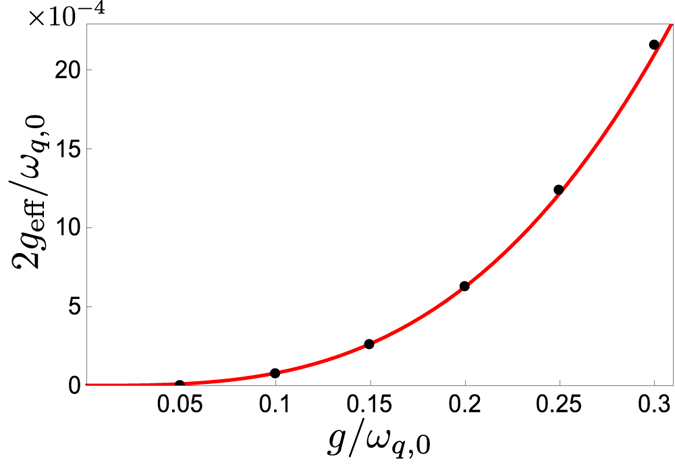
Comparison of analytical (red curve) and numerical (black dots) results for the effective coupling between the states |1, 0, *g*〉 and |0, 2, *e*〉. The graph shows the minimum energy splitting 2*g*_eff_/*ω*_*q*,0_ as a function of *g*/*ω*_*q*,0_, using the same parameters as in Fig. 6.

The two-photon frequency conversion can be performed either by adiabatic transfer or by tuning the qubit into resonance for half a Rabi oscillation period, as explained in the section on single-photon frequency conversion. In the first approach, one adiabatically tunes the qubit energy to follow one of the energy levels shown in Fig. 6(a). A simulation of the second approach, including decoherence, is shown in Fig. 6(b). The timescale for these processes is set by the effective coupling. In Fig. 7, we show that the expression for the effective coupling given in Eq. (8) remains a good approximation up to at least *g* = 0.3*ω*_*q*,0_ for the parameters used in Fig. 6.

### The |1, 0, *e*〉 ↔ |0, 2, *g*〉 process

**Figure 8:**
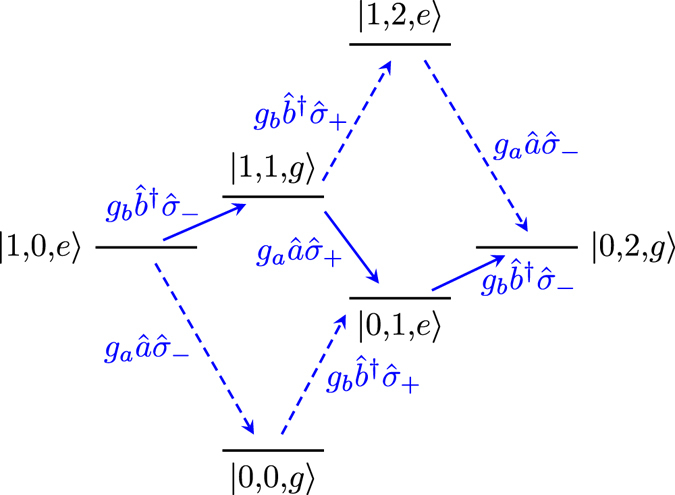
The lowest-order processes contributing to a transition between |1, 0, *e*〉 and |0, 2, *g*〉 in the quantum Rabi model. The transitions that do not conserve excitation number are shown as dashed blue lines and the excitation-number-conserving transitions are shown as solid blue lines. The label of each line is the term in Eq. (6) that gives rise to that transition. The parameters *ω*_*a*_ = 3*ω*_*q*_ and *ω*_*b*_ = 2*ω*_*q*_ were used to set the positions of the energy levels.

For the process |1, 0, *e*〉 ↔ |0, 2, *g*〉, we show in Fig. 8 the virtual transitions from the quantum Rabi Hamiltonian that contribute to lowest order. We note that this process conserves the excitation number, which means that there is a path between the states that can be realised using only terms from the Jaynes–Cummings (JC) Hamiltonian^99^ (solid arrows in the figure). Below, we analyse the effective coupling both for the full quantum Rabi Hamiltonian and for the JC Hamiltonian. Usually, the JC Hamiltonian is obtained by performing the RWA on the quantum Rabi Hamiltonian when *g* ≪ *ω*_*a*/*b*/*q*_, in which case the low coupling strength would make it very challenging to observe the frequency conversion process. However, we note that a circuit QED setup has been proposed where the pure JC Hamiltonian with ultrastrong coupling can be realised^100^.

### Quantum Rabi Hamiltonian

Retaining only the states shown in Fig. 8, we can write the quantum Rabi Hamiltonian from Eq. (6) on matrix form as

10 $${\hat{H}}_{{\rm{R}}}=(\begin{array}{cccccc}-\frac{{\omega }_{q}}{2} & {g}_{b} & {g}_{a} & 0 & 0 & 0\\ {g}_{b} & {\omega }_{b}+\frac{{\omega }_{q}}{2} & 0 & \sqrt{2}{g}_{b} & {g}_{a} & 0\\ {g}_{a} & 0 & {\omega }_{a}+\frac{{\omega }_{q}}{2} & 0 & {g}_{b} & 0\\ 0 & \sqrt{2}{g}_{b} & 0 & 2{\omega }_{b}-\frac{{\omega }_{q}}{2} & 0 & {g}_{a}\\ 0 & {g}_{a} & {g}_{b} & 0 & {\omega }_{a}+{\omega }_{b}-\frac{{\omega }_{q}}{2} & \sqrt{2}{g}_{b}\\ 0 & 0 & 0 & {g}_{a} & \sqrt{2}{g}_{b} & {\omega }_{a}+2{\omega }_{b}+\frac{{\omega }_{q}}{2}\end{array}),$$

where the states are ordered as |0, 0, *g*〉, |0, 1, *e*〉, |1, 0, *e*〉, |0, 2, *g*〉, |1, 1, *g*〉, and |1, 2, *e*〉. As in previous calculations, we can perform adiabatic elimination close to the resonance, which in this case is *ω*_*a*_ + *ω*_*q*_ ≈ 2*ω*_*b*_. The details of the elimination are given in the Supplementary Information. The result is an effective coupling between the states |1, 0, *e*〉 and |0, 2, *g*〉 with magnitude

11 $${g}_{{\rm{eff}}}=\frac{\sqrt{2}{g}^{3}[2{\omega }_{b}({\omega }_{a}-2{\omega }_{b})-{g}^{2}]}{2{\omega }_{b}^{2}{({\omega }_{a}-{\omega }_{b})}^{2}+{g}^{2}{\omega }_{b}\mathrm{(5}{\omega }_{b}-3{\omega }_{a})+{g}^{4}}$$

on resonance. We have set *g*_*a*_ = *g*_*b*_ ≡ *g* to simplify the expression slightly. Note that this expression for the coupling is actually exactly the same as the one for the process |1, 0, *g*〉 ↔ |0, 2, *e*〉 given in Eq. (8). Even though the two processes use different intermediate states, the truncated Hamiltonians in Eqs (7) and (10) only differ in the sign of *ω*_*q*_. Since *ω*_*q*_ is replaced on resonance by (*ω*_*a*_ − 2*ω*_*b*_) in the first case and by (2*ω*_*b*_ − *ω*_*a*_) in the second case, the formula for the effective coupling ends up being the same in both cases. The two cases still differ, however. For example, while the limit *ω*_*a*_ → *ω*_*b*_, which enhances the coupling, could not occur for the process |1, 0, *g*〉 ↔ |0, 2, *e*〉, it is possible for |1, 0, *e*〉 ↔ |0, 2, *g*〉. However, in this limit the approximations behind the adiabatic elimination break down, since the states |1, 1, *g*〉 and |0, 1, *e*〉 would also be on resonance and become populated.

**Figure 9:**
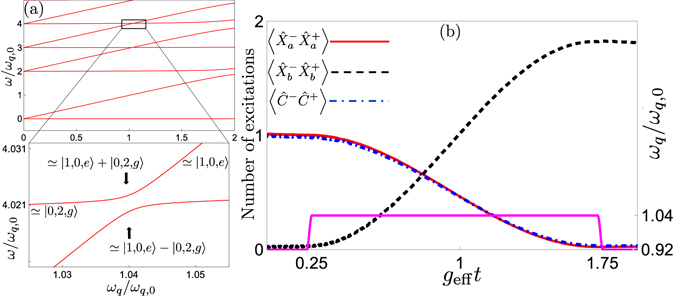
Two-photon frequency conversion via transitions between |1, 0, *e*〉 and |0, 2, *g*〉. (a) The energy levels of our system, given in Eq. (6), plotted as a function of the qubit frequency *ω*_*q*_, using the parameters *g*_*a*_ = *g*_*b*_ = 0.125*ω*_*q*,0_, *ω*_*a*_ = 3*ω*_*q*,0_, and *ω*_*b*_ = 2*ω*_*q*,0_, where the reference point *ω*_*q*,0_ is set such that *ω*_*a*_ + *ω*_*q*,0_ = 2*ω*_*b*_. In the zoom-in, close to the resonance *ω*_*a*_ + *ω*_*q*_ = 2*ω*_*b*_, we see the anticrossing between |1, 0, *e*〉 and |0, 2, *g*〉 with the splitting 2*g*_eff_ given by Eq. (11). Up-conversion of a photon pair into a single photon, or down-conversion of a single photon into a photon pair, can be achieved by adiabatically tuning *ω*_*q*_ to follow one of the energy levels in the figure from |0, 2, *g*〉 to |1, 0, *e*〉, or vice versa. (b) A rapid frequency conversion can be achieved by starting in |1, 0, *e*〉 or |0, 2, *g*〉, far from the resonance *ω*_*a*_ + *ω*_*q*_ = 2*ω*_*b*_, tuning the qubit frequency (pink solid curve, a smoothed step function as explained in Fig. 3) into resonance for half a Rabi period (*π*/2*g*_eff_) and then tuning it out of resonance again. The figure shows the number of excitations in the two resonators (red solid curve for *a*, black dashed-dotted curve for *b*) and the qubit (blue dashed curve) during such a process, including decoherence in the form of relaxation from the resonators and the qubit. The parameters used for the decoherence are *κ*_*a*_ = *κ*_*b*_ = *γ* = 4 × 10^−5^ *ω*_*q*,0_.

The two-photon frequency conversion can again be performed either by adiabatic transfer or by tuning the qubit into resonance for half a Rabi oscillation period, as explained in the section on single-photon frequency conversion. The energy levels to follow in the first approach are plotted in Fig. 9(a) and a simulation of the second approach, including decoherence, is shown in Fig. 9(b).

### Jaynes–Cummings Hamiltonian

For completeness, we calculate the effective coupling using only the JC Hamiltonian for two resonators and one qubit, i.e., we eliminate the non-excitation-conserving terms in the quantum Rabi Hamiltonian of Eq. (6), giving

12 $${\hat{H}}_{{\rm{JC}}}={\omega }_{a}{\hat{a}}^{\dagger }\hat{a}+{\omega }_{b}{\hat{b}}^{\dagger }\hat{b}+\frac{{\omega }_{q}}{2}{\hat{\sigma }}_{z}+{g}_{a}(\hat{a}{\hat{\sigma }}_{+}+{\hat{a}}^{\dagger }{\hat{\sigma }}_{-})+{g}_{b}(\hat{b}{\hat{\sigma }}_{+}+{\hat{b}}^{\dagger }{\hat{\sigma }}_{-}).$$

Retaining only the states connected by solid arrows in Fig. 8, we can write the Hamiltonian from Eq. (12) on matrix form as

13 $${\hat{H}}_{{\rm{JC}}}=(\begin{array}{cccc}{\omega }_{b}+\frac{{\omega }_{q}}{2} & 0 & \sqrt{2}{g}_{b} & {g}_{a}\\ 0 & {\omega }_{a}+\frac{{\omega }_{q}}{2} & 0 & {g}_{b}\\ \sqrt{2}{g}_{b} & 0 & 2{\omega }_{b}-\frac{{\omega }_{q}}{2} & 0\\ {g}_{a} & {g}_{b} & 0 & {\omega }_{a}+{\omega }_{b}-\frac{{\omega }_{q}}{2}\end{array}),$$

where the states are ordered as |0, 1, *e*〉, |1, 0, *e*〉, |0, 2, *g*〉, and |1, 1, *g*〉. Again, we perform adiabatic elimination close to the resonance *ω*_*a*_ + *ω*_*q*_ ≈ 2*ω*_*b*_. The details of the elimination are given in the Supplementary Information. The result is an effective coupling between the states |1, 0, *e*〉 and |0, 2, *g*〉 with magnitude

14 $${g}_{{\rm{eff}}}=-\frac{\sqrt{2}{g}_{a}{g}_{b}^{2}}{{g}_{a}^{2}+{({\omega }_{a}-{\omega }_{b})}^{2}}$$

on resonance. Just as for the other two-photon frequency-conversion processes, the coupling scales like *g* ^3^/*ω* ^2^ to leading order. In fact, Eq. (14) is a good approximation to Eq. (11), since the path given by the JC terms (solid lines) in Fig. 8 is far less detuned in energy from the initial and final states than all the other paths and thus gives the largest contribution to the result in Eq. (11). The remarks on the limit *ω*_*a*_ → *ω*_*b*_ given after Eq. (11) apply here as well. The schemes for implementing the frequency conversion are already given in Fig. 9.

**Figure 10:**
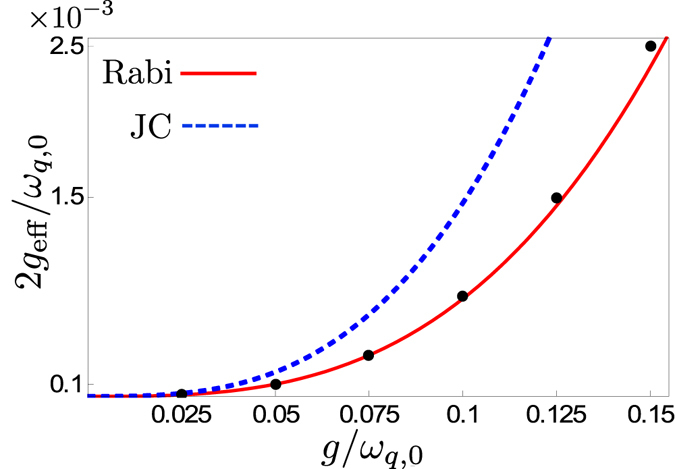
Comparison of analytical (JC Hamiltonian [dashed blue curve] and quantum Rabi Hamiltonian [solid red curve]) and numerical (black dots) results for the effective coupling between the states |1, 0, *e*〉 and |0, 2, *g*〉. The graph shows the minimum energy splitting 2*g*_eff_/*ω*_*q*,0_ as a function of *g*/*ω*_*q*,0_, using the same parameters as in Fig. 9.

In Fig. 10, we compare the results from Eqs (11) and (14) with a full numerical calculation using the quantum Rabi Hamiltonian. The contribution from the JC part dominates the coupling up until around *g*_*a*_ = *g*_*b*_ = *g* = 0.03*ω*_*q*,0_ and gives a good approximation until then. For higher values of the coupling, using the approximation from the quantum Rabi Hamiltonian instead works fine up until around *g*_*a*_ = *g*_*b*_ = *g* = 0.15*ω*_*q*,0_. It is interesting to note that a pure JC Hamiltonian in the USC regime would give higher effective coupling for this frequency-conversion process than the quantum Rabi Hamiltonian.

## Discussion

We have shown how a system consisting of two resonators ultrastrongly coupled to a qubit can be used to realise a variety of frequency-conversion processes. In particular, we have shown how to convert a single photon into another photon of either higher or lower frequency, as well as how to convert a single photon into a photon pair and vice versa. All these processes are deterministic, can be implemented within a single setup, and do not require any external drives. The conversion is controlled by tuning the frequency of the qubit to and from values that make the desired transitions resonant.

Given the recent advances in USC circuit QED, we believe that our proposal can be implemented in such a setup. Indeed, two resonators have already been ultrastrongly coupled to a superconducting flux qubit^49^. Also, our proposal does not require very high coupling strengths. We only need that *g* ^2^/*ω* is appreciable (larger than the relevant decoherence rates) to realise single-photon frequency conversion; multi-photon frequency conversion can be demonstrated if *g* ^3^/*ω* ^2^ is large enough.

A straightforward extension of the current work is to extend the calculations to processes with more photons in the second resonator or to add more resonators to the setup. Some of these possibilities are discussed in ref. 71, where we explore analogies of nonlinear optics in USC systems, including the fact that the processes in the current work can be considered analogies of Raman and hyper-Raman scattering if the qubit is thought of as playing the role of a phonon. More general three-wave mixing, such as |1, 0, 0, *e*〉 ↔ |0, 1, 1, *g*〉, or third-harmonic and -subharmonic generation such as |1, 0, *e*〉 ↔ |0, 3, *g*〉, are examples of schemes that can be considered, but it must be kept in mind that higher-order processes with more photons involved will have lower effective coupling strengths. Another direction for future work is to investigate how the precise qubit control of the frequency-conversion processes discussed here can be used to prepare photon bundles^101^ or interesting quantum superposition states with photons of different frequencies, a topic currently being explored in several frequency ranges^26, 27, 102^.

## Electronic supplementary material

Supplementary Information
