# Supplementary material for: Frequency conversion in ultrastrong cavity QED
Source: Sci Rep. 2017 Jul 13;7:5313. doi: 10.1038/s41598-017-04225-3 (PMC5509682; doi:10.1038/s41598-017-04225-3)
Supplement: Supplementary file 1 — Supplementary Information [file 41598_2017_4225_MOESM1_ESM.pdf]

Supplementary Information for
Frequency conversion in ultrastrong cavity QED

Anton Frisk Kockum,[1] Vincenzo Macrì,[2,1] Luigi Garziano,[3,2,1] Salvatore Savasta,[2,1] and Franco Nori[1,4]

[1]Center for Emergent Matter Science, RIKEN, Saitama 351-0198, Japan
[2]Dipartimento di Scienze Matematiche e Informatiche, Scienze Fisiche e Scienze della Terra, Università di Messina, I-98166 Messina, Italy
[3]School of Physics and Astronomy, University of Southampton, Southampton, SO17 1BJ, United Kingdom
[4]Physics Department, The University of Michigan, Ann Arbor, Michigan 48109-1040, USA

S1. ANALYTICAL CALCULATIONS OF CONVERSION RATES

In this Supplementary Information, we present the full adiabatic-elimination calculations for the effective couplings in the three processes considered in this article: $|1,0,g\rangle \leftrightarrow |0,1,e\rangle$, $|1,0,g\rangle \leftrightarrow |0,2,e\rangle$, and $|1,0,e\rangle \leftrightarrow |0,2,g\rangle$. For each case, we compare the analytical results with numerical simulations to determine in what parameter regimes the analytical calculations constitute a good approximation.

A. $|1,0,g\rangle \leftrightarrow |0,1,e\rangle$

Starting from the truncated Hamiltonian in Eq. (3), we move to a frame rotating with $(\omega_a - \frac{\omega_q}{2})$, i.e., subtracting $(\omega_a - \frac{\omega_q}{2})$ from the diagonal of the Hamiltonian, giving

$$\hat{H} = \begin{pmatrix} -\omega_a & 0 & -g_a \sin\theta & g_b \cos\theta & 0 & 0 \\ 0 & -(\omega_a - \omega_q) & g_a \cos\theta & g_b \sin\theta & 0 & 0 \\ -g_a \sin\theta & g_a \cos\theta & 0 & 0 & -g_b \sin\theta & g_b \cos\theta \\ g_b \cos\theta & g_b \sin\theta & 0 & \omega_b + \omega_q - \omega_a & g_a \cos\theta & g_a \sin\theta \\ 0 & 0 & -g_b \sin\theta & g_a \cos\theta & \omega_b & 0 \\ 0 & 0 & g_b \cos\theta & g_a \sin\theta & 0 & \omega_b + \omega_q \end{pmatrix}. \tag{S1}$$

Denoting the amplitudes of the six states by c_1–c_6, respectively, the Schrödinger equation gives

$$i\dot{c}_1 = -\omega_a c_1 - g_a \sin\theta c_3 + g_b \cos\theta c_4, \tag{S2}$$
$$i\dot{c}_2 = -(\omega_a - \omega_q) c_2 + g_a \cos\theta c_3 + g_b \sin\theta c_4, \tag{S3}$$
$$i\dot{c}_3 = -g_a \sin\theta c_1 + g_a \cos\theta c_2 - g_b \sin\theta c_5 + g_b \cos\theta c_6, \tag{S4}$$
$$i\dot{c}_4 = (\omega_b + \omega_q - \omega_a) c_4 + g_b \cos\theta c_1 + g_b \sin\theta c_2 + g_a \cos\theta c_5 + g_a \sin\theta c_6, \tag{S5}$$
$$i\dot{c}_5 = \omega_b c_5 - g_b \sin\theta c_3 + g_a \cos\theta c_4, \tag{S6}$$
$$i\dot{c}_6 = (\omega_b + \omega_q) c_6 + g_b \cos\theta c_3 + g_a \sin\theta c_4. \tag{S7}$$

Assuming that $\omega_a \approx \omega_b + \omega_q$, and that $g_a, g_b \ll \omega_a, \omega_b, |\omega_a - \omega_q|, \omega_b + \omega_q$, we can adiabatically eliminate the four intermediate levels (their population will not change significantly), i.e., set $\dot{c}_1 = \dot{c}_2 = \dot{c}_5 = \dot{c}_6 = 0$. This gives

$$c_1 = -\frac{g_a \sin\theta}{\omega_a} c_3 + \frac{g_b \cos\theta}{\omega_a} c_4, \tag{S8}$$
$$c_2 = \frac{g_a \cos\theta}{\omega_a - \omega_q} c_3 + \frac{g_b \sin\theta}{\omega_a - \omega_q} c_4, \tag{S9}$$
$$c_5 = \frac{g_b \sin\theta}{\omega_b} c_3 - \frac{g_a \cos\theta}{\omega_b} c_4, \tag{S10}$$
$$c_6 = -\frac{g_b \cos\theta}{\omega_b + \omega_q} c_3 - \frac{g_a \sin\theta}{\omega_b + \omega_q} c_4, \tag{S11}$$

which we then insert into the equations for c_3 and c_4 to arrive at

$$\begin{aligned} i\dot{c}_3 = &\left(\frac{g_a^2 \sin^2\theta}{\omega_a} + \frac{g_a^2 \cos^2\theta}{\omega_a - \omega_q} - \frac{g_b^2 \sin^2\theta}{\omega_b} - \frac{g_b^2 \cos^2\theta}{\omega_b + \omega_q} \right) c_3 \\ &+ \frac{1}{2} g_a g_b \sin 2\theta \left(\frac{1}{\omega_a - \omega_q} + \frac{1}{\omega_b} - \frac{1}{\omega_a} - \frac{1}{\omega_b + \omega_q} \right) c_4, \end{aligned} \tag{S12}$$

$$i\dot{c}_4 = \frac{1}{2} g_a g_b \sin 2\theta \left(\frac{1}{\omega_a - \omega_q} + \frac{1}{\omega_b} - \frac{1}{\omega_a} - \frac{1}{\omega_b + \omega_q} \right) c_3 + \left(\omega_b + \omega_q - \omega_a + \frac{g_b^2 \cos^2\theta}{\omega_a} + \frac{g_b^2 \sin^2\theta}{\omega_a - \omega_q} - \frac{g_a^2 \cos^2\theta}{\omega_b} - \frac{g_a \sin\theta}{\omega_b + \omega_q} \right) c_4. \tag{S13}$$

While the energy level shifts in these equations are not final (they can be affected by processes involving more energy levels), the effective coupling rate between $|1,0,g\rangle$ and $|0,1,e\rangle$ is shown to be

$$g_{\text{eff}} = \frac{1}{2} g_a g_b \sin 2\theta \left(\frac{1}{\omega_a - \omega_q} + \frac{1}{\omega_b} - \frac{1}{\omega_a} - \frac{1}{\omega_b + \omega_q} \right). \tag{S14}$$

Assuming that we are exactly on resonance, the qubit frequency can be eliminated from this expression using $\omega_q = \omega_a - \omega_b$, leading to

$$g_{\text{eff}} = g_a g_b \sin 2\theta \left(\frac{1}{\omega_b} - \frac{1}{\omega_a} \right) = \frac{g_a g_b (\omega_a - \omega_b) \sin 2\theta}{\omega_a \omega_b}, \tag{S15}$$

the first part of which is given in Eq. (5). We note that the result agrees with the perturbation-theory calculations performed in Ref. [1]. In general, the adiabatic elimination is more exact, but for a second-order process the result for the effective coupling is the same with both methods.

B. $|1,0,g\rangle \leftrightarrow |0,2,e\rangle$

Starting from the truncated Hamiltonian in Eq. (7), we move to a frame rotating with $(\omega_a - \frac{\omega_q}{2})$, i.e., subtracting $(\omega_a - \frac{\omega_q}{2})$ from the diagonal of the Hamiltonian, giving

$$\hat{H}_{\text{R}} = \begin{pmatrix} \omega_q - \omega_a & g_b & g_a & 0 & 0 & 0 \\ g_b & \omega_b - \omega_a & 0 & \sqrt{2} g_b & g_a & 0 \\ g_a & 0 & 0 & 0 & g_b & 0 \\ 0 & \sqrt{2} g_b & 0 & 2\omega_b - \omega_a + \omega_q & 0 & g_a \\ 0 & g_a & g_b & 0 & \omega_b + \omega_q & \sqrt{2} g_b \\ 0 & 0 & 0 & g_a & \sqrt{2} g_b & 2\omega_b \end{pmatrix}. \tag{S16}$$

Denoting the amplitudes of the six states by c_1–c_6, the Schrödinger equation gives

$$i\dot{c}_1 = (\omega_q - \omega_a) c_1 + g_b c_2 + g_a c_3, \tag{S17}$$
$$i\dot{c}_2 = (\omega_b - \omega_a) c_2 + g_b c_1 + \sqrt{2} g_b c_4 + g_a c_5, \tag{S18}$$
$$i\dot{c}_3 = g_a c_1 + g_b c_5, \tag{S19}$$
$$i\dot{c}_4 = (2\omega_b - \omega_a + \omega_q) c_4 + \sqrt{2} g_b c_2 + g_a c_6, \tag{S20}$$
$$i\dot{c}_5 = (\omega_b + \omega_q) c_5 + g_a c_2 + g_b c_3 + \sqrt{2} g_b c_6, \tag{S21}$$
$$i\dot{c}_6 = 2\omega_b c_6 + g_a c_4 + \sqrt{2} g_b c_5. \tag{S22}$$

Assuming that $\omega_a \approx 2\omega_b + \omega_q$, and that $g_a, g_b \ll \omega_b + \omega_q, |\omega_b - \omega_q|, |\omega_a - \omega_q|$, we can adiabatically eliminate the four intermediate levels, i.e., set $\dot{c}_1 = \dot{c}_2 = \dot{c}_5 = \dot{c}_6 = 0$. This gives

$$i\dot{c}_3 = \frac{g_a^4 \omega_b + g_a^2 \left[\omega_b (\omega_a - \omega_b)^2 - g_b^2 (\omega_a + \omega_b) \right] + g_b^2 \omega_b \left[g_b^2 + 2\omega_b (\omega_b - \omega_a) \right]}{2\omega_b^2 \left[g_a^2 + (\omega_a - \omega_b)^2 \right] + g_b^4 + 3 g_b^2 \omega_b (\omega_b - \omega_a)} c_3 + \frac{g_a g_b^2 \left[g_a^2 - 3 g_b^2 + 4\omega_b (\omega_a - 2\omega_b) \right]}{\sqrt{2} \left\{ 2\omega_b^2 \left[g_a^2 + (\omega_a - \omega_b)^2 \right] + g_b^4 + 3 g_b^2 \omega_b (\omega_b - \omega_a) \right\}} c_4, \tag{S23}$$

where we simplified the expressions somewhat by setting $\omega_q = \omega_a - 2\omega_b$. While the energy level shift in this equation is not final (they can be affected by processes involving more energy levels), the effective coupling rate between $|1,0,g\rangle$ and $|0,2,e\rangle$ is shown to be

$$g_{\text{eff}} = \frac{g_a g_b^2 \left[g_a^2 - 3 g_b^2 + 4\omega_b (\omega_a - 2\omega_b) \right]}{\sqrt{2} \left\{ 2\omega_b^2 \left[g_a^2 + (\omega_a - \omega_b)^2 \right] + g_b^4 + 3 g_b^2 \omega_b (\omega_b - \omega_a) \right\}}. \tag{S24}$$

Setting $g_a = g_b \equiv g$, this reduces to

$$g_{\text{eff}} = \frac{\sqrt{2}g^3\left[2\omega_b(\omega_a - 2\omega_b) - g^2\right]}{2\omega_b^2(\omega_a - \omega_b)^2 + g^2\omega_b(5\omega_b - 3\omega_a) + g^4}, \tag{S25}$$

which is Eq. (8). We can simplify the expression for the coupling further by only keeping terms to leading order in g/ω; the result is

$$g_{\text{eff}} = \frac{\sqrt{2}g^3\left(\omega_a - 2\omega_b\right)}{\omega_b\left(\omega_a - \omega_b\right)^2}, \tag{S26}$$

which is Eq. (9). This agrees with the perturbation-theory calculation in Ref. [1], which only captures the leading-order term.

C. $|1,0,e\rangle \leftrightarrow |0,2,g\rangle$

For the process $|1,0,e\rangle \leftrightarrow |0,2,g\rangle$, we perform adiabatic elimination starting from both the quantum Rabi Hamiltonian and the JC Hamiltonian.

1. *Quantum Rabi Hamiltonian*

Starting from the truncated Hamiltonian in Eq. (10), we move to a frame rotating with $(\omega_a + \frac{\omega_q}{2})$, i.e., subtracting $(\omega_a + \frac{\omega_q}{2})$ from the diagonal of the Hamiltonian, giving

$$\hat{H}_{\text{R}} = \begin{pmatrix} -\omega_a - \omega_q & g_b & g_a & 0 & 0 & 0 \\ g_b & \omega_b - \omega_a & 0 & \sqrt{2}g_b & g_a & 0 \\ g_a & 0 & 0 & 0 & g_b & 0 \\ 0 & \sqrt{2}g_b & 0 & 2\omega_b - \omega_a - \omega_q & 0 & g_a \\ 0 & g_a & g_b & 0 & \omega_b - \omega_q & \sqrt{2}g_b \\ 0 & 0 & 0 & g_a & \sqrt{2}g_b & 2\omega_b \end{pmatrix}. \tag{S27}$$

Denoting the amplitudes of the six states by c_1–c_6, the Schrödinger equation gives

$$i\dot{c}_1 = -(\omega_a + \omega_q)c_1 + g_b c_2 + g_a c_3, \tag{S28}$$
$$i\dot{c}_2 = (\omega_b - \omega_a)c_2 + g_b c_1 + \sqrt{2}g_b c_4 + g_a c_5, \tag{S29}$$
$$i\dot{c}_3 = g_a c_1 + g_b c_5, \tag{S30}$$
$$i\dot{c}_4 = (2\omega_b - \omega_a - \omega_q)c_4 + \sqrt{2}g_b c_2 + g_a c_6, \tag{S31}$$
$$i\dot{c}_5 = (\omega_b - \omega_q)c_5 + g_a c_2 + g_b c_3 + \sqrt{2}g_b c_6, \tag{S32}$$
$$i\dot{c}_6 = 2\omega_b c_6 + g_a c_4 + \sqrt{2}g_b c_5. \tag{S33}$$

Assuming that $\omega_a + \omega_q \approx 2\omega_b$, and that $g_a, g_b \ll |\omega_b - \omega_q|, \omega_b + \omega_q, \omega_a + \omega_q, |\omega_a - \omega_q|$, we can adiabatically eliminate the four intermediate levels, i.e., set $\dot{c}_1 = \dot{c}_2 = \dot{c}_5 = \dot{c}_6 = 0$. This gives

$$\begin{aligned} i\dot{c}_3 = &\frac{g_a^4\omega_b + g_a^2\left[\omega_b(\omega_a - \omega_b)^2 - g_b^2(\omega_a + \omega_b)\right] + g_b^2\omega_b\left[g_b^2 + 2\omega_b(\omega_b - \omega_a)\right]}{2\omega_b^2\left[g_a^2 + (\omega_a - \omega_b)^2\right] + g_b^4 + 3g_b^2\omega_b(\omega_b - \omega_a)} c_3 \\ &+ \frac{g_a g_b^2\left[g_a^2 - 3g_b^2 + 4\omega_b(\omega_a - 2\omega_b)\right]}{\sqrt{2}\left\{2\omega_b^2\left[g_a^2 + (\omega_a - \omega_b)^2\right] + g_b^4 + 3g_b^2\omega_b(\omega_b - \omega_a)\right\}} c_4, \end{aligned} \tag{S34}$$

where we simplified the expressions somewhat by setting $\omega_q = 2\omega_b - \omega_a$. While the energy level shift in this equation is not final (it can be affected by processes involving more energy levels), the effective coupling rate between $|1,0,e\rangle$ and $|0,2,g\rangle$ is shown to be

$$g_{\text{eff}} = \frac{g_a g_b^2\left[g_a^2 - 3g_b^2 + 4\omega_b(\omega_a - 2\omega_b)\right]}{\sqrt{2}\left\{2\omega_b^2\left[g_a^2 + (\omega_a - \omega_b)^2\right] + g_b^4 + 3g_b^2\omega_b(\omega_b - \omega_a)\right\}}. \tag{S35}$$

Setting $g_a = g_b \equiv g$, this reduces to

$$g_{\rm eff} = \frac{\sqrt{2}g^3\left[2\omega_b(\omega_a - 2\omega_b) - g^2\right]}{2\omega_b^2(\omega_a - \omega_b)^2 + g^2\omega_b(5\omega_b - 3\omega_a) + g^4}, \tag{S36}$$

which is Eq. (11). As noted in the main text, this is equal to the coupling for the case $|1,0,g\rangle \leftrightarrow |0,2,e\rangle$, but other values of ω_a and ω_b are permitted in this case. In particular, the coupling can be increased by letting $\omega_a \to \omega_b$, but the approximations we have used here break down when $|\omega_a - \omega_b|$ becomes comparable to g. Again, the result agrees with the perturbation-theory calculation in Ref. [1], which only captures the leading-order term.

2. Jaynes–Cummings Hamiltonian

Starting from the truncated Hamiltonian in Eq. (13), we move to a frame rotating with $(\omega_a + \frac{\omega_q}{2})$, i.e., subtracting $(\omega_a + \frac{\omega_q}{2})$ from the diagonal of the Hamiltonian, giving

$$\hat{H}_{\rm JC} = \begin{pmatrix} \omega_b - \omega_a & 0 & \sqrt{2}g_b & g_a \\ 0 & 0 & 0 & g_b \\ \sqrt{2}g_b & 0 & 2\omega_b - \omega_a - \omega_q & 0 \\ g_a & g_b & 0 & \omega_b - \omega_q \end{pmatrix}. \tag{S37}$$

Denoting the amplitudes of the four states by c_1–c_4, the Schrödinger equation gives

$$i\dot{c}_1 = (\omega_b - \omega_a)c_1 + \sqrt{2}g_b c_3 + g_a c_4, \tag{S38}$$

$$i\dot{c}_2 = g_b c_4, \tag{S39}$$

$$i\dot{c}_3 = (2\omega_b - \omega_a - \omega_q)c_3 + \sqrt{2}g_b c_1, \tag{S40}$$

$$i\dot{c}_4 = (\omega_b - \omega_q)c_4 + g_a c_1 + g_b c_2. \tag{S41}$$

Assuming that $\omega_a + \omega_q \approx 2\omega_b$, and that $g_a, g_b \ll \omega_a, \omega_b, \omega_q$, we can adiabatically eliminate the two intermediate levels, i.e., set $\dot{c}_1 = \dot{c}_4 = 0$. This gives

$$i\dot{c}_2 = -\frac{g_b^2(\omega_a - \omega_b)}{g_a^2 + (\omega_a - \omega_b)^2}c_2 - \frac{\sqrt{2}g_a g_b^2}{g_a^2 + (\omega_a - \omega_b)^2}c_3, \tag{S42}$$

where we set $\omega_q = 2\omega_b - \omega_a$. While the energy level shift in this equation is not final (it can be affected by processes involving more energy levels), the effective coupling rate between $|1,0,e\rangle$ and $|0,2,g\rangle$ is shown to be

$$g_{\rm eff} = -\frac{\sqrt{2}g_a g_b^2}{g_a^2 + (\omega_a - \omega_b)^2}, \tag{S43}$$

which is Eq. (14). Setting $g_a = g_b \equiv g$, this reduces to

$$g_{\rm eff} = -\frac{\sqrt{2}g^3}{g^2 + (\omega_a - \omega_b)^2}, \tag{S44}$$

which to leading order in g/ω becomes

$$g_{\rm eff} = -\frac{\sqrt{2}g^3}{(\omega_a - \omega_b)^2}, \tag{S45}$$

agreeing with the perturbation-theory calculation of Ref. [1].

[1] A. F. Kockum, A. Miranowicz, V. Macrì, S. Savasta, and F. Nori, "Deterministic quantum nonlinear optics with single atoms and virtual photons," arXiv:1701.05038 (2017), arXiv:1701.05038.
